# Does antibiotic use accelerate or retard cutaneous repair? A systematic review in animal models

**DOI:** 10.1371/journal.pone.0223511

**Published:** 2019-10-10

**Authors:** Luciana Schulthais Altoé, Raul Santos Alves, Mariáurea Matias Sarandy, Mônica Morais-Santos, Rômulo Dias Novaes, Reggiani Vilela Gonçalves

**Affiliations:** 1 Departament of General Biology, Federal University of Viçosa, Viçosa, Minas Gerais, Brazil; 2 Departament of Animal Biology, Federal University of Viçosa, Viçosa, Minas Gerais, Brazil; 3 Departament of Structural Biology, Federal University of Alfenas, Alfenas, Minas Gerais, Brazil; University of Mississippi Medical Center, UNITED STATES

## Abstract

**Background:**

The presence of infections is one of the main factors that leads to delays in healing or non-closure of cutaneous wounds. Although the goal of antibiotic use is to treat or prevent infection, there is currently no agreement on the effectiveness of these products.

**Aim:**

The aim of this study was to evaluate the efficacy of antibiotic use during the healing process of skin wounds in animal models not intentionally infected, as well as to analyze the advances and limitations of the studies carried out in this field.

**Main methods:**

This systematic review was performed according to the PRISMA guidelines, using a structured search on the MedLine (PubMed) and Scopus platforms to retrieve studies published until August 29, 2018, 13:35p.m. The studies included were limited to those that used excision or incision wound models and that were not intentionally infected. The data for the animal models, antibiotic used, and the main results of the studies were extracted, and compared where possible. Bias analysis and methodological quality assessments were examined through the SYRCLE’s Risk of Bias tool.

**Key findings:**

Twenty-seven studies were selected. Overall, the effects of the antibiotic on the wound decreased inflammatory cell infiltration and promoted an increased number of fibroblasts, extracellular matrix constituents, re-epithelialization and tissue strength. A great deal of important information about the methodology was not presented, such as: the statistical analysis used, the animal model (sex and age), antibiotic dosage, blinding and randomization of the animals chosen.

**Significance:**

Based on the results found, we believe that antibiotic therapy can be considered a viable alternative for the treatment of cutaneous wounds. However, current evidence obtained from the methodological quality analysis points towards a high risk of bias. This is due to the incomplete characterization of the experimental design and treatment protocol, which compromises the reproducibility of the studies.

## Introduction

Skin plays an important role in protecting the body against aggressive agents, forming a barrier that prevents the entry and proliferation of pathogens [1]. When a tissue injury occurs, due to the action of microorganisms or trauma, the body initiates a series of complex events, aiming to reestablish the structure of the damaged skin tissue. The repair process can be divided into four stages: hemostasis, inflammation, proliferation and remodeling [2]. During hemostasis, platelets aggregate at the site of the lesion and initiate the process of blood coagulation, which also promotes vascular hemostasis and releases chemotactic factors that stimulate the migration and activation of immune cells that will be important for the next phase of the process [3]. In the next phase, leukocyte infiltration into the injured site occurs and these cells release proinflammatory cytokines, such as interleukins (IL1, IL6), tumor necrosis factor-α (TNF-α), and interferon gamma (IFN-γ); In addition, antimicrobial substances are also released, such as reactive oxygen species and proteases, that clean the wound and prepare the tissue for deposition of the extracellular matrix. In addition to inflammatory cells, fibroblasts are also attracted to the lesion site at this stage, which are mainly responsible for the synthesis of collagen and elastin [4,5]. The next phase is known as proliferative, characterized by the formation of granulation tissue, rich in blood vessels and collagen type III fibers [6]. This tissue is fragile and poorly resistant to traction, but it is the necessary basis for definitive tissue deposition. In addition, epithelium regenerates at this stage [7]. Remodeling is the final phase, where the fragile tissue is replaced by a strong tissue rich in collagen type I with a large number of cross-links forming bundles of fibers that give the new tissue mechanical strength [4,8].

The coordination and regulation of cellular, humoral and molecular processes can lead to perfect tissue regeneration [9]. However, factors such as the presence of infections, advanced patient age and metabolic disorders, can cause an imbalance in the repair process preventing adequate progression and wound closure [9,10]. Currently, a high prevalence of cutaneous lesions has been observed in elderly and diabetic populations, with a significant increase in the incidence of chronic wounds worldwide [10,11]. Often disguised as a comorbidity, chronic wounds represent a silent epidemic that worsens the patient’s quality of life [12] and causes a significant financial burden on the public health system, since treatment for chronic wounds is expensive and time-consuming [13]. For example, in the United States of America, the Medicare dataset on all wound categories, including acute and chronic, estimated expenditures ranging from $28.1 to $96.8 billion on wound treatment [14]. Another report from Wales estimated a prevalence of 6% of chronic wounds at a cost of 5.5% for National Health Service (NHS) [15]. Therefore, the economic impact generated by wounds is a concern. In addition to the costs involved in treating and managing the wounds, when not treated effectively, cutaneous lesions do not close and can progress to severe sepsis, amputation and even lead to patient mortality [16].

The progression of a wound to an infected state and consequent chronification of the lesion is determined by the ability of the host to generate an effective immune response, in addition to the amount of pathogens that come into contact with the injured tissue [17]. The exposed subcutaneous tissue provides a moist, warm and nutritious environment that is favorable to the proliferation of a wide variety of microorganisms [18]. To avoid colonization by pathogens and consequent chronification of lesions, there is a broad spectrum of therapies available on the market, but their effects on the repair process are still unclear. In this context, antibiotics have been growing as a therapeutic alternative. Some studies support the routine use of antibiotics in wound management due to their favoring cellular and vascular proliferation, thereby accelerating the closure of cutaneous lesions [19,20], as well as being effective in reducing infection [18]. Therefore, knowing that one of the major complications of wound healing is infection, the correct use of antibiotics can speed up wound healing and significantly reduce health care costs [21]. However, there is still a discussion regarding the effectiveness of using antibiotics in the treatment of not intentionally infected wounds due to their possible cytotoxic effect on fibroblasts and keratinocytes [22,23]. Given the uncertainties and controversies surrounding the role of antibiotics in treating cutaneous wounds, this review aimed to analyze the evidence regarding the use of antibiotics in the repair of cutaneous wounds by experimental incision or excision, in not intentionally infected animal models. Furthermore, the study evaluated the role of antibiotics in wound management and the relevance of the treatment. Methodological quality was also analyzed, together with the advances and limitations of these studies, identifying the main sources of bias.

## Materials and methods

### Focus question

The main question to be answered in this systematic review was: Does the use of antibiotics accelerate or slow cutaneous repair in animal models? Second, what are the main methodological parameters used to evaluate the evolution of the repair process in not intentionally infected animal models?

### Search strategy

**Fig 1:**
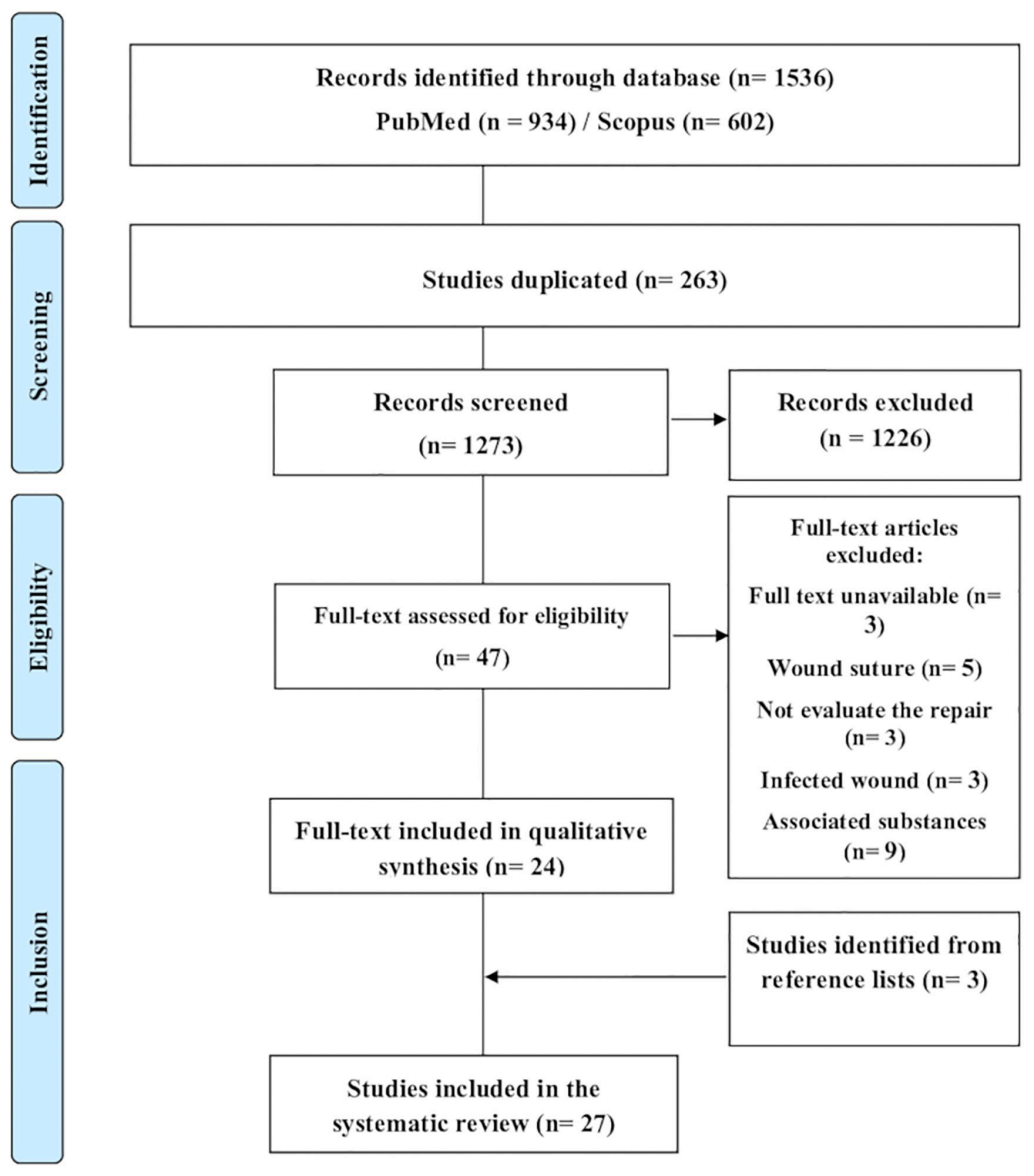
PRISMA diagram. Different phases of selection of studies for conducting qualitative and quantitative analyses. Flow diagram of the systematic review literature search results. Based on ‘Preferred Reporting Items for Systematic Reviews and Meta-Analyses: The PRISMA Statement’. http://www.prisma-statement.org. From: Moher D, Liberati A, Tetzlaff J, Altman DG, The PRISMA Group (2009).

This systematic review followed the Preferred Reporting Items for Systematic Reviews and Meta-Analyses (PRISMA) guidelines [24] (Fig 1). The studies were selected through an advanced search on the platforms PubMed and Scopus, on August 29, 2018 13:35 p.m. Based on two search parameters, we devised a comprehensive search strategy for the retrieval of all relevant studies: (i) direct searches in electronic databases, and (ii) indirect screening of reference lists from all studies identified in the direct searches. For all databases, the search filters were based on four complementary levels: (i) animals, (ii) wound healing, (iii) skin and (iv) antibiotics. Search filters were initially developed for PubMed, the search algorithms [MeSH Terms] and [TIAB] were applied, to identify indexed records and those recently published in indexing processes, respectively. To detect all *in vivo* animal model studies in PubMed, a standardized and optimized animal filter was obtained [25]. The terms used to search on PubMed were adapted for the selection of Scopus publications, and the “animal model” filter is provided by the site itself. No date limit was applied. Languages were restricted to articles in English, Portuguese and Spanish. The abstracts of all articles chosen were interpreted to identify potentially eligible articles. A consensus process that was informed by evidence, was used to develop a 27-item checklist (S1 Table).

Two reviewers (LSA and RSA) conducted the literature search, removed duplicate articles, and screened titles and abstracts with respect to eligibility criteria. After initial screening, full-text articles of potentially relevant studies were independently assessed for eligibility by two reviewers (LSA and RSA). The kappa test was done for the selection and data extraction (kappa = 0.839). Selections were then compared, and inconsistencies were resolved in consultation with three other reviewers (MMS, RDN and RVG).

### Inclusion and exclusion criteria

Only original studies investigating the use of antibiotics in the cutaneous repair process of non-sutured incisional and excisional animal wound models were included. To identify the antibiotics, the List of Critically Important Antimicrobials for Human Medicine (WHO CIA List) guide developed by World Health Organization (WHO), the fifth revision of the WHO CIA List, published by WHO in 2016 was used [26]. We excluded from the review studies that used antimicrobial drugs not described by the aforementioned guide, studies that used only *in vitro* or *ex vivo* experimental models, studies with no full-text available, secondary studies (literature reviews, letters to the editor, case studies, comments and editorials), studies with plant species or peptides, studies with other organs or tissues, studies in diabetic animals and wounds resulting from burns. Two reviewers (LSA and RSA) manually searched reference lists of studies selected in the previous step independently to find additional relevant articles.

### Data extraction

Three independent reviewers (LSA, RSA and MMS) extracted the essential data grouped into four descriptive levels as follows: (i) publication characteristics (authors, date of publication, and country); (ii) characteristics of the animal models (species, sex, age, and weight); (iii) experimental interventions (asepsis, biopsy day, dermal wound instrument, wound size, number of wounds per animal, anesthesia); (iv) information about antibiotic treatment (name of the antibiotic used, dose, frequency of administration, route of administration, pharmaceutical form and treatment in the control group); (v) the results from groups treated with antibiotics for all the studies were pooled (synthesis of extracellular matrix components, neovascularization, wound strength, time to wound closure). Any inconsistencies regarding the extracted data were resolved during discussions with two additional reviewers (RVG and RDN).

### Assessment of risk of bias in included studies

To assess the risk of bias in the studies included, SYRCLE’s Risk of Bias (RoB) tool, designed specifically for animal studies, was used [25]. The following methodological domains based on RoB were evaluated. Consider selection bias: “Was the allocation sequence adequately generated and applied?”, “Were the groups similar at baseline or were they adjusted for confounders in the analysis?”, “Was the allocation to the different groups adequately concealed?”; Consider performance bias: “Were the animals randomly housed during the experiment?”, “Were the caregivers and/or researchers blinded regarding which intervention each animal received during the experiment?”; Consider detection bias: “Were animals selected at random for outcome assessment?”, “Was the outcome assessor blinded?”; Considers attrition bias: “Were incomplete outcome data adequately addressed?”; Considers reporting bias: “Are reports of the study free of selective outcome reporting?”; Considers other biases: “Was the study apparently free of other problems that could result in high risk of bias?”; The overall study quality indicators: “Was randomization at any level the experiment indicated?” and “Was it stated that the experiment was blinded at any level?”. The items in the RoB tool were scored with “yes” (low risk of bias); “no” (high risk of bias); or “unclear” (indicating that the item was not reported, and therefore, the risk of bias was unknown).

## Results

### Characteristics of publications

The initial research resulted in 1536 studies, with 934 from PubMed and 602 from Scopus. Out of these, 263 were excluded because they were duplicate studies. After reading the titles and abstracts, 47 relevant studies were selected and read in full, and their references checked. Finally, 27 studies fully met the inclusion criteria and were included in the systematic review. The process of selecting articles is shown in a flowchart (Fig 1). The filters applied in each database and the flowchart indicating the search structure are shown in S2 Table.

The 27 studies were conducted in eight different countries: the United States of America (40.47%, n = 11), South Korea, India (14.81%, n = 4 each), Taiwan (11.11%, n = 3), China (7.41%, n = 2), Turkey, South Africa and Australia (3.70%, n = 1 each).

An absence of an animal ethics committee was observed in 25.93% of studies (n = 7) and statistical analysis of the individual studies was performed in 77.78% of studies (n = 21), while 22.22% of the studies (n = 6) did not report any statistical comparison of the data (S3 Table).

### Characteristics of experimental animals

As shown in S3 Table, rats were the main animal model used (55.56%, n = 15), followed by pigs (18.52%, n = 5), mice (11.11%, n = 3), horses and rabbits (5.56%, n = 2 each). The proportion of sex of animals was 44.44% male (n = 12), 22.22% female (n = 6) and 3.70% both (n = 1). Eight studies did not specify the sex of the animals (29.63%). The age of the animals was only specified in 22.22% of the studies (n = 6). In 14.81% of studies (n = 4) adult or young animals were described without a specific age while 62.96% did not report this information. Body weight data was specified in most studies (85.19%, n = 23) while 14.81% of studies omitted this information (n = 4). 25.93% of studies omitted the total number of animals in the experiment (n = 7). In the studies where this information was reported, 10% used up to 5 animals (n = 2), 40% ranged from 6 to 15 animals (n = 8), 30% ranged from 16 to 25 animals (n = 6) and 20% used more than 25 animals (n = 4) (S3 Table).

### Wound characteristics

59.26% of the studies (n = 16) reported the type of asepsis performed prior to wounding, with ethanol being the substance most commonly used (43.75%, n = 7), followed by povidone-iodine (PI) (18.75%, n = 3). Combinations such as saline, PI + ethanol, PI + saline, chlorhexidine gluconate (CHX), CHX + saline and CHX + saline + ethanol were used in 4.55% of studies (n = 1 each). The day of the experiment when the biopsy was performed was specified in 74.07% of studies (n = 20), whereas 18.52% did not biopsy the wound (n = 5) and 7.41% did not indicate the day of the procedure (n = 2). The instrument used in wounding was reported in 51.85% (n = 14) of studies. 35.71% of studies (n = 5) used scalpels, 21.43% used punch biopsy (n = 3), 14.29% used scissors (n = 2), 14.29% used dermatome (n = 2) and 7.14% used punch biopsy + scalpel or dermatome + scissor (n = 1 each). All studies reported the wound size while the number of wounds made was reported in 85.19% (n = 23). 25% of studies made two wounds on the same animal (n = 6), 20.83% one or four wounds (n = 5 each), 8.33% six wounds (n = 2) and 4.17% made 12, 16, 20, 36, 45 or 150 wounds per animal (n = 1 each). The anesthetic specifications were reported in 96.30% (n = 26) of the studies with the most commonly cited being: ketamine + xylazine and pentobarbital (11.54%, n = 3 each) (S4 Table).

### Antibiotic characteristics

#### Related information in studies

**Fig 2:**
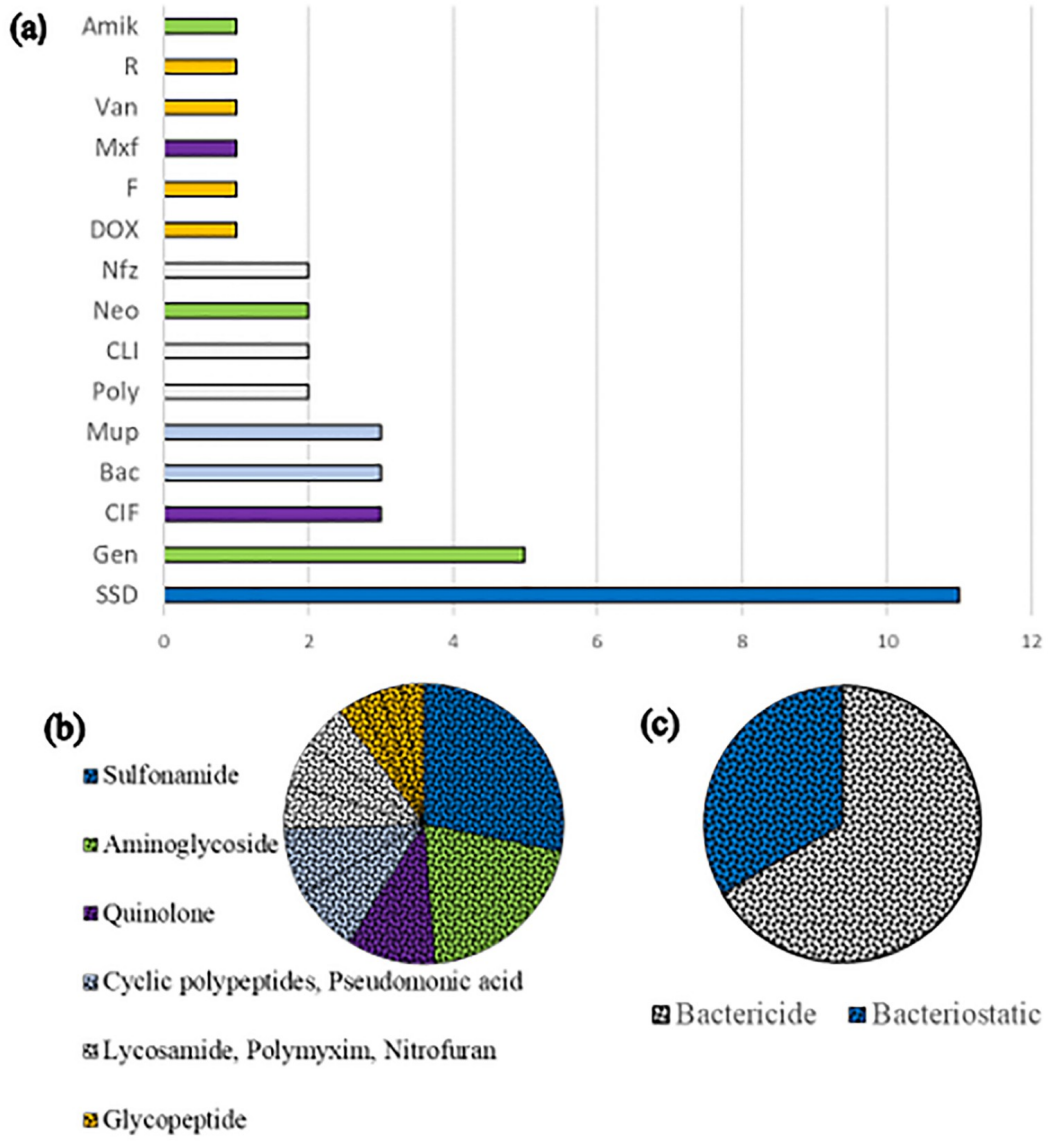
Characteristic of the antibiotic used in the studies of this systematic review that evaluated the effect of the antibiotic on the healing of not intentionally infected wounds. (a) antibiotics used in the studies, (b) classes of antibiotics. Van = vancomycin, Amik = Amikacin, Gen = gentamicin, Mxf = Moxifloxacin, Nfz = Nitrofurazone, SSD = silver sulfadiazine, SA = sodium alginate, CLI = clindamycin, CIF = ciprofloxacin, Dex = dexamethasone, Mup = mupirocin, Bac = bacitracin, Poly = polymyxin B, R = rifamycin, F = fusidic acid, Neo = neomycin, DOX = doxycycline.

Fifteen types of antibiotic were observed in the selected studies, with the most frequently used being silver sulfadiazine (28.2%, n = 11), followed by gentamicin (12.8%, n = 5), ciprofloxacin, mupirocin and bacitracin (7.7%, n = 3 each). The antibiotics clindamycin, neomycin, nitrofurazon and polymycin b were used in 2 studies each (5.1%). The antibiotics amikacin, doxycycline, fusidic acid, moxifloxacin, rifamicin, and vancomycin were only used in one study each (2.6% each) (Fig 2a).

74.07% (n = 20) of studies reported the concentration of antibiotic used while 25.93% (n = 7) of studies did not indicate this information. The frequency of antibiotic administration was reported in 25 studies (92.59%). The most frequent administration timeframe was a single day (48%, n = 12), 28% ranged between two to seven days (n = 7), 16% between eight to fourteen days (n = 4) and 8% longer than 15 days (n = 2). The most common administration route was topical (88.89%, n = 24), followed by oral (7.41%, n = 2) and intravenous (3.7%, n = 1). The main mode of administration of the antibiotic was through curative (48.15%, n = 13) on the injured skin, while 25.93% (n = 7) administered the antibiotic in a cream, 14.81% (n = 4) in an ointment, 7.41% administered it in powdered form dissolved in the drinking water of the animals and 3.7% (n = 1) administered it in liquid form, by means of injection.

Most studies used more than one control group and the interventions performed in these groups were: untreated (30.95%, n = 13), vehicle (28.57%, n = 12), gauze (16.67%, n 7), commercial product (9.54%, n = 4), saline (4.76%, n = 2), Ky lubricant^®^, bandaged, Vaseline gauze, hydrocolloid, povidone-iodine (PI) (2.38%, n = 1 each) (Table 1).

**Table 1:** Description of the main characteristics of the antibiotic treatments used in the studies of this systematic review that evaluated the effect of the antibiotic on the healing of not intentionally infected wounds.

Antibiotic						
**Animal model: Rat**						
**Reference**	**Antibiotic**	**Classe**	**Concentration**	**Frequency (d)**	**Format**	**Control**
Leitch, et al. 1993 [27]	Silver sulfadiazine (SSD)	Sulfonamide	?	?	Cream	Untreated
Heggers et al. 1995 [28]	Silver sulfadiazine (SSD)/ mupirocin (Mup)/ clindamycin (CLI)	Sulfonamide/ pseudomonic acids/ lincosamide	1–2%	14	Cream/ ointment	Untreated
Choi et al. 1999 [29]	Silver sulfadiazine (SSD)	Sulfonamide	0.4 mg/cm^2^	1	Curative	Vaseline gauze
Muller et al. 2003 [30]	Silver sulfadiazine (SSD)	Sulfonamide	0.5/ 1%	14	Cream	Sal/ vehicle
Kim, et al. 2008 [31]	Clindamycin (CLI)	Lincosamide	?	1	Curative	Gauze
Kim, et al. 2008 [32]	Nitrofurazone (nfz)	Nitrofuran	?	1	Curative	Vehicle
Simpson, et al. 2008 [33]	Bacitracin (Bac)+ neomycin (Neo)+ polymyxin B (Poly)	Cyclic polypeptide/ aminoglycoside/ polymyxin	?	9	Ointment	Untreated
Hwang et al. 2010 [34]	Gentamicin (gen)	Aminoglycoside	0.1%	1	Curative	Gauze/ commercial product/ vehicle
Lin et al. 2010 [35]	Gentamicin (gen)	Aminoglycoside	0,50 mg/ mL	1	Curative	Gauze
Huang et al. 2012 [36]	Gentamicin (gen)	Aminoglycosides	0.05%	1	Curative	Gauze/ hydrocolloid dressing
Gurel et al. 2013 [37]	Rifamycin (R)/ fusidic acid (F)	Ansamycin/ steroid antibacterial	0.1 cm^3^/ 0.25 g	7	?	Sal
Mittal and Kumar 2014 [38]	Gentamicin (gen)	Aminoglycoside	590 μg/ mg	1	Curative	Untreated
Princely et al. 2015 [39]	Gentamicin (Gen)	Aminoglycoside	?	1	Curative	Vehicle/ PI
Fu et al. 2016 [40]	Moxifloxacin (Mxf)	Quinolone	2%	1	Curative	Commercial product/ vehicle/ untreated
Li et al. 2017 [41]	Ciprofloxacin (CIF)	Quinolone	0,9%	7	Curative	Gauze
**Animal model: Pig**						
**Reference**	**Antibiotic**	**Classe**	**Concentration**	**Frequency (d)**	**Format**	**Control**
Geronemus, et al. 1979 [42]	Bacitracin (Bac)+ neomycin (Neo)+ polymyxin B (Poly)/ nitrofurazone (Nfz) / silver sulfadiazine (SSD)	Cyclic polypeptides/ aminoglycoside/ polymyxin/ nitrofuran / sulfonamide	?	6	Ointment	Untreated/ vehicle
Watcher and Wheeland 1989 [43]	Bacitracin (Bac)/ silver sulfadiazine (SSD)/ mupirocin (Mup)	Cyclic polypeptides/ sulfonamide/ pseudomonic acid	1–2%	27	ointment/ cream	K-Y lubricant/ untreated
Singer, et al. 1999 [44]	Silver sulfadiazine (SSD)	Sulfonamide	?	4	?	Gauze
Faucher, et al. 2010 [45]	Silver sulfadiazine (SSD)	Sulfonamide	1%	1	Curative	Gauze/ vehicle/ untreated
Theunissen et al. 2016 [46]	Mupirocin (Mup)/ silver sulfadiazine (SSD)	Pseudomonic acid/ sulfonamide	1–2%	28	Cream	Untreated
**Animal model: Mice**						
**Reference**	**Antibiotic**	**Classe**	**Concentration**	**Frequency (d)**	**Format**	**Control**
Hebda et al. 2003 [47]	Doxycycline (DOX)	Tetracycline	2 mg/ml	3	Powder	Vehicle
Zhang et al. 2015 [48]	Vancomycin (Van)	Glycopeptides	4 mg/ml	12	Powder	Vehicle
Tummalapalli et al. 2016 [49]	Ciprofloxacin (CIF)	Quinolone	0.5–2.5%	1	Curative	Commercial product/ vehicle/ untreated
**Animal model: Rabbit**						
**Reference**	**Antibiotic**	**Classe**	**Concentration**	**Frequency (d)**	**Format**	**Control**
Kataria et al. 2014 [50]	Ciprofloxacin (CIF)	Quinolone	32–35 mg/ mL	1	Curative	Commercial product/ vehicle/ untreated
Qian, et al. 2017 [51]	Silver sulfadiazine (SSD)	Sulfonamide	0.01–1%	2/7	Cream	Vehicle
**Animal model: Horse**						
**Reference**	**Antibiotic**	**Classe**	**Concentration**	**Frequency (d)**	**Format**	**Control**
Berry and Sullins 2003 [52]	Silver sulfadiazine (SSD)	Sulfonamide	1%	?	Cream	Bandaged/ untreated
Edwards-Milewski et al. 2016 [53]	Amikacin (amik)	Aminoglycoside	5 mg/ kg	3	Liquid	Untreated

PI = povidone-iodine, Sal = saline.

#### Antibiotic identification

The antibiotics were divided into 12 antibiotic classes (Fig 2b). Most antibiotics present bactericidal effects (66.7%, n = 8), while others present bacteriostatic action (33.3%, n = 4) (Fig 2a).

The action mechanism for most antibiotics is inhibition of bacterial protein synthesis (38.5%, n = 15), followed by inhibition of folic acid metabolism required for the synthesis of bacterial DNA and RNA (28.2%, n = 11), by action on bacterial nucleic acids, either by inhibiting their synthesis or causing damage (17.9%, n = 7), by inhibiting bacterial cell wall synthesis (10.3%, n = 4) or by alteration of the bacterial cell cytoplasmic membrane (5.1%, n = 2).

The class of antibiotic most commonly used in the studies was sulfonamide (28.2%, n = 11), which was represented only by silver sulfadiazine. Results in which healing was favored (n = 3) showed an increase in the rate of re-epithelialization, number of fibroblasts, matrix components and reduction of wound area. Some studies also showed no effect on healing time for this antibiotic (n = 6) and that it can lead to repair delays (n = 2), with a reduction in rates of re-epithelialization and an increase of wound half-life and rupture strength.

The second most used class in the studies was the class of aminoglycosides (20.5%, n = 8), which was represented by gentamicin, amikacin and neomycin. In studies where this class led to increased healing rates (n = 5), there was increased collagen production, re-epithelization, and proliferation of fibroblasts and blood vessels. In addition, there was a reduction of inflammation and wound area. In studies where the healing time did not differ from the control (n = 1), the production of extracellular matrix and the area and contraction of the wound did not change, however there was an increase in inflammatory cells. There was no delay in healing in any study. An association of the aminoglycoside, cyclic polypeptides and polymyxim classes was observed in two studies. The association of these three antibiotics led to improved healing, with increased re-epithelialization (n = 1). In another study the healing time was unaffected (n = 1), showing the same rates of re-epithelialization and wound contraction as the control group.

The third most commonly used class of antibiotics was the quinolone class (10.3%, n = 4), and was represented by ciprofloxacin and moxifloxacin. When healing was favored (n = 3), the area of the wound and inflammation were reduced, as well as presenting an increase in the extracellular matrix. In the study in which the healing time was not affected (n = 1), there was an increase in collagen synthesis and organization, increased vascularization and number of fibroblasts. No healing delay was observed in any study.

The class of pseudomonic acid, represented only by mupirocin, and the cyclic polypeptides class, represented only by bacitracin, were analyzed in three studies each (7.7%). In the pseudomonic acid class, when the healing time was reduced (n = 1) there was an increase in granulation tissue, fibroblasts, extracellular matrix formation and re-epithelialization rate. In the study where the healing time did not differ from the control group (n = 1), the re-epithelialization rate was unaffected, but there were delays in wound contraction. When this class of antibiotics presented delays in healing time (n = 1), the rupture force of the wound increased. The cyclic polypeptide class was used in only one study without being associated with other antibiotics and did not alter the healing time in relation to the control. In addition, a smaller wound contraction was observed, even though greater re-epithelialization was also noted.

The antibiotic classes lycosamide, represented only by clindamycin, and nitrofuran, represented only by nitrofurazone, were analyzed in two studies each (5.1%). In the study in which lycosamide led to decreased healing time (n = 1), there was a reduction of inflammation and wound area and increased granulation tissue. In the study where a delay in healing time was observed (n = 1), lyncosamide increased the rupture force of the wound. In the study that applied Nitrofuran, a reduction in healing time (n = 1) was observed, with a consequent reduction of wound area and inflammatory infiltrate. However, it was also seen that this class led to a decrease in re-epithelialization and consequent delays in healing (n = 1).

The classes tetracycline, glycopeptide, ansamycin and antibacterial steroid were used in one study each (2.6%). The tetracycline class was represented by doxycycline and did not show changes in healing time, but presented increased collagen organization and consequently, increased rupture force of the wound force rupture. The glycopeptide class was represented by vancomycin, leading to delayed healing time and decreased expression of RegIIIγ, the secreted C-type lectin. The class ansamycin represented by rifamycin led to lower re-epithelialization, inflammatory infiltrate, vascularization and fibroblast numbers and as a result, healing was faster than in the control group. The antibacterial steroid class was represented by fusidic acid with a delay in healing time being observed due to the greater intensity of fibroblast accumulation, which caused a longer proliferative phase, with less vascularization and inflammation.

#### Main outcomes

In most of the studies, the process of cutaneous repair was accelerated by antibiotic treatment. Even in the studies in which the antibiotics did not reduce healing time in relation to the control groups, antibiotic treatments generally led to positive outcomes, increasing extracellular matrix components and the rupture force of the wound. In the antibiotic formulations found in the studies, we observed that 45.71% (n = 16) of antibiotics tested presented a reduction in healing time, 34.29% (n = 12) did not alter the healing time and, 20.0% (n = 7) led to a slower healing time.

**Fig 3:**
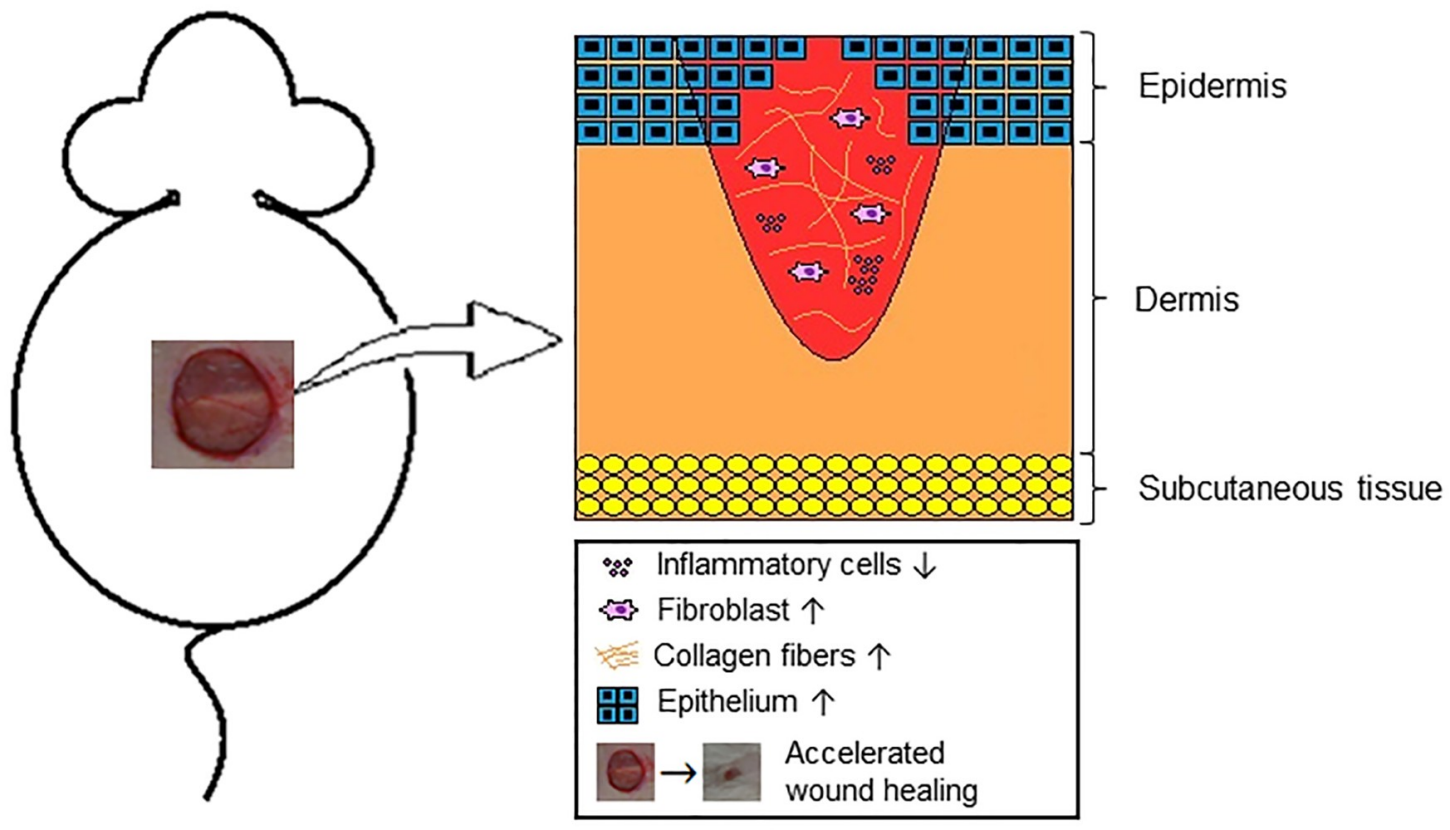
A schematic diagram of the general action of antibiotics in healing of not intentionally infected cutaneous wounds in animal models.

In studies where healing time was shortened, an increase in fibroblasts, extracellular matrix components and re-epithelization were observed, as well as reductions in inflammatory infiltrate and the wound area. In the studies where healing time was unaffected by the administration of antibiotics, the treatment favored collagen organization and consequently an increase in wound force, neovascularization and an increase in fibroblasts. The results described above are shown in Fig 3. Considering the articles that described the antibiotics, and which presented a longer healing time, the main results that explain this finding were an increase in wound half-life, wound area and a reduction in re-epithelialization. Additionally, there was an increase in tissue resistance, because the rupture force of the wound increased (Table 2).

**Table 2:** Main results of the action of the classes of antibiotics on wound healing in animal models not infected. The results were separated according to healing time in relation to the control.

Class	Healing time	Measure outcomes
Sulfonamide	Reduction (n = 3)	↑ Reepithelialization, fibroblasts, ECM ↓ Wound area
	Similar (n = 6)	= Reepithelialization, wound area, wound contraction
	Increase (n = 2)	↑ Wound half-life, rupture strength ↓ Reepithelialization
Aminoglycoside	Reduction (n = 5)	↑ Fibroblasts, reepithelialization, ECM, blood vessels ↓ Wound area, inflammatory cells
	Similar (n = 1)	= Wound area, Wound contraction ↑ inflammatory cells
Quinolone	Reduction (n = 3)	↑ ECM ↓ Wound area, inflammatory cells
	Similar (n = 1)	↑ ECM, blood vessels, fibroblasts
Pseudomonic acid	Reduction (n = 1)	↑ Granulation tissue, fibroblasts, ECM, reepithelialization
	Similar (n = 1)	= Reepithelialization ↓ Wound contraction
	Increase (n = 1)	↑ Wound area, rupture strength, wound half-life
Lycosamide	Reduction (n = 1)	↑ Granulation tissue ↓ Wound area, inflammatory cells
	Increase (n = 1)	↑ Wound area, rupture strength, wound half-life
Nitrofuran	Reduction (n = 1)	↓ Wound area, inflammatory cells
	Increase (n = 1)	↓ Reepithelialization
Tetracycline	Similar (n = 1)	= Wound area, reepithelialization ↑ Rupture strength
Glycopeptide	Increase (n = 1)	↑ Wound area ↓ Expression of RegIIIγ
Ansamycin	Reduction (n = 1)	↓ Reepithelialization, inflammatory cells, blood vessels, fibroblasts
Steroid antibacterial	Increase (n = 1)	= ECM ↑ Fibroblasts ↓ Reepithelialization, inflammatory cells, blood vessels
Cyclic polypeptides	Similar (n = 1)	↑ Reepithelialization ↓ Wound contraction
Cyclic polypeptides + Aminoglycoside + Polymyxim teroid antibacterial	Reduction (n = 1)	↑ Reepithelialization
	Similar (n = 1)	= Reepithelialization, wound area, wound contraction

ECM = extracellular matrix

### Risk of bias and methodological quality assessments

**Fig 4:**
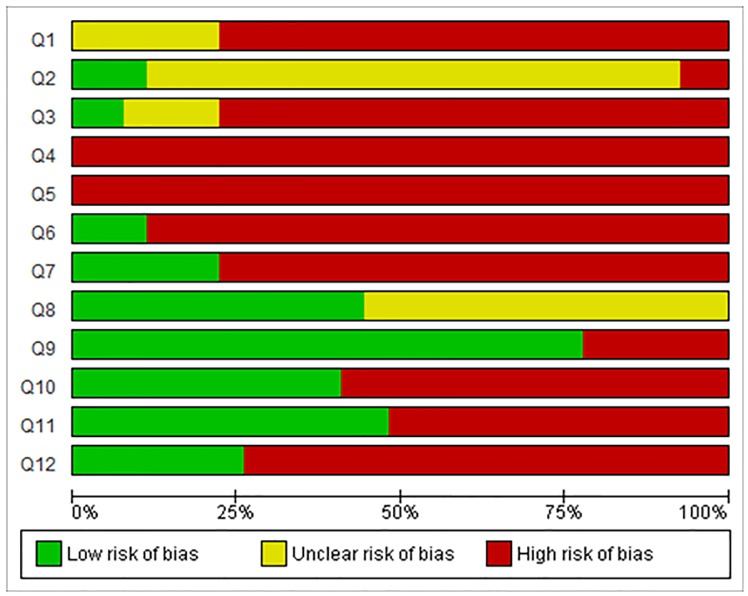
Results for the risk of bias and methodological quality indicators for all studies included in this systematic review that evaluated the effect of the antibiotic on the healing of not intentionally infected wounds. The items in the Systematic Review Centre for Laboratory Animal Experimentation (SYRCLE) Risk of Bias assessment (Q1–Q10) were scored with “yes” indicating low risk of bias, “no” indicating high risk of bias, or “unclear” indicating that the item was not reported, resulting in an unknown risk of bias. Q1–Q3 consider selection bias, Q4–Q5 consider performance bias, Q6–Q7 consider detection bias, Q8 considers attrition bias, Q9 considers reporting bias, and Q10 considers other biases. The overall study quality indicators (Q11–Q12) were scored with “yes” when reported or “no” when not reported. Q, question. Q1: Was the allocation sequence adequately generated and applied?; Q2: Were the groups similar at baseline or were they adjusted for confounders in the analysis?; Q3: Was the allocation to the different groups adequately concealed?; Q4: Were the animals randomly housed during the experiment?; Q5: Were the caregivers and/or investigators blinded from knowledge regarding which intervention each animal received during the experiment?; Q6: Were animals selected at random for outcome assessment?; Q7: Was the outcome assessor blinded?; Q8: Were incomplete outcome data adequately addressed?; Q9: Are reports of the study free of selective outcome reporting?; Q10: Was the study apparently free of other problems that could result in high risk of bias?; Q11: Was it stated whether the experiment was randomized at any level?; Q12: Was it stated whether the experiment was blinded at any level?

**Fig 5:**
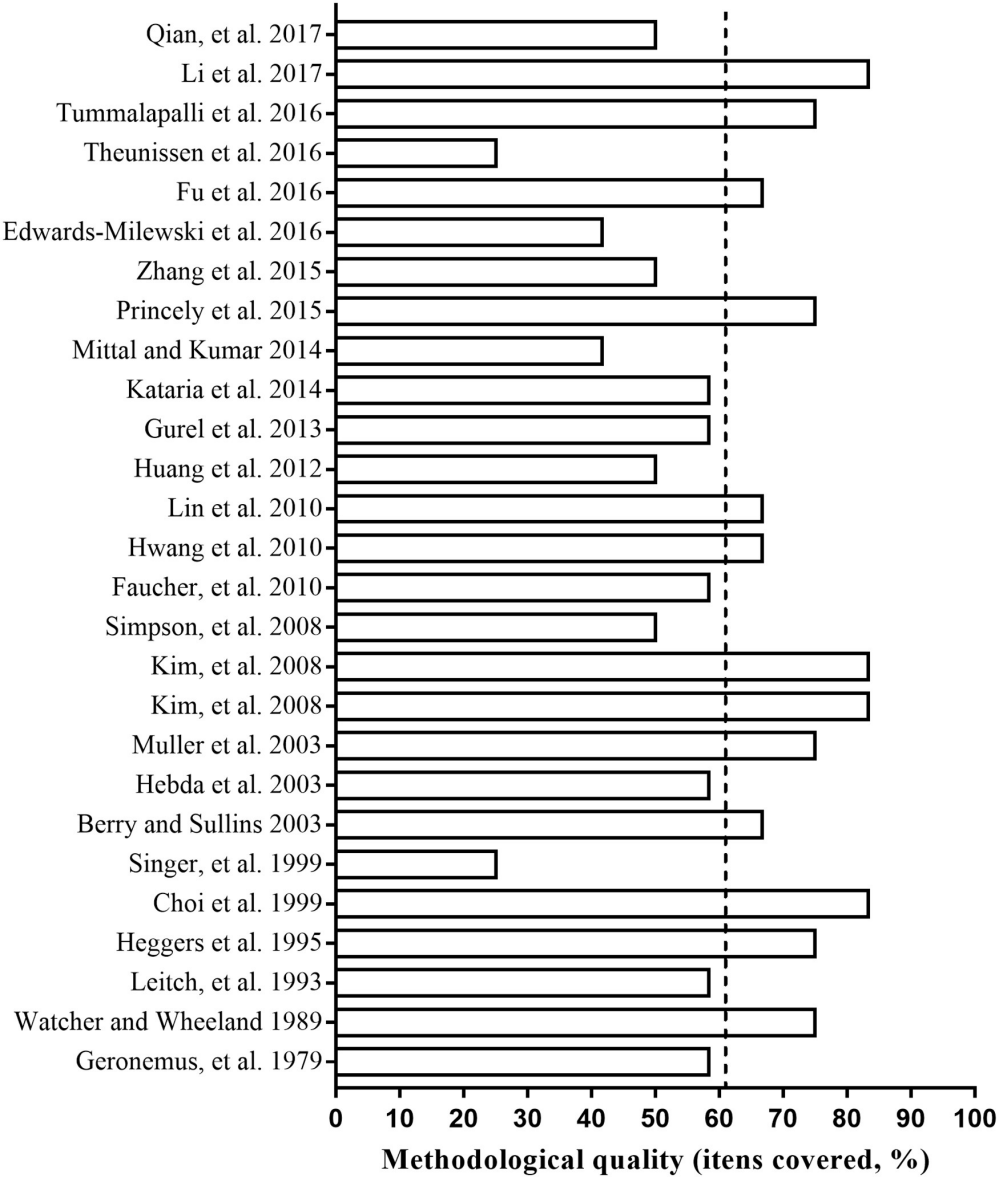
Analysis of high risk of bias of each study included in the review: Year of publication versus high risk of bias. Based in the Systematic Review Centre for Laboratory Animal Experimentation (SYRCLE) Risk of Bias.

The detailed results for the analysis of bias are shown in the Fig 4. No studies fulfilled all the methodological criteria analyzed. In relation to selection bias, the sequence generation process was not reported in 77.78% studies (n = 21). In terms of the animal’s characteristics, that is, their similarity to each other (Q2), 22 studies (81.48%) did not report this information clearly. 21 studies (77.78%) did not report information regarding the allocation concealment (Q3). None of the articles reported random housing or blinding of caregivers (Q4 and Q5, respectively) and as such, the outcome was evaluated as presenting high risk of bias. 24 studies (88.89%) did not report random outcome assessment for detection bias, for relevant outcome measures (Q6). In addition, the outcome assessor was not reported to have been blinded in 21 studies (77.78%; Q7). 15 studies (55.56%; Q8) showed incomplete outcome data. 6 studies (22.22%) presented a high risk for reporting bias (Q9). In addition, 16 studies (59.26%; Q10) presented other potential sources of bias. Two quality indicators were used to assess the methodological quality of the studies. 51.85% of the studies (n = 14) reported no randomization at any level of the experiment (Q11). 74.07% of studies (n = 20; Q12) did not report blinding. The analysis of the individual studies found no relation between risk of bias and the year the studies were published (Fig 5). Thus, the most recent studies do not describe the methodological quality criteria, in comparison with older studies.

## Discussion

In our study, we conducted a systematic revision to investigate antibiotic use in the healing of not intentionally infected cutaneous wounds. Our results showed that antibiotic use can accelerate the process of cutaneous healing, reducing the wound area and inflammatory infiltration, as well as increasing the number of fibroblasts, the synthesis of extracellular matrix components (MEC), the formation of epithelial tissue and the force of wound closure. However, a small number of studies showed that antibiotic therapies can also negatively affect healing, leading to slower wound closure. These findings, given that the inadequate use of antibiotics can lead to an increase in bacterial resistance [54], mean that the choice of antibiotic therapy for treating cutaneous wounds should be given careful consideration. Currently, the global consumption of antibiotics is increasing considerably. Between 2000 and 2015, there was a 65% increase in the use of these compounds [55], with the USA standing out as the biggest consumer of antibiotics in the world, when taking into consideration other high income countries [55]. This finding was confirmed by our research, as the greater part of the studies observed in this revision were carried out in the United States, followed by South Korea and India.

During wound healing, there are some steps that are extremely important for the formation of a strong scar free of infection. In this context, we can highlight the inflammatory phase, which is characterized by the recruitment of the innate immune system, which acts against attacks by invasive pathogens, helping to remove dead tissue [56, 57]. However, prolonged inflammation is prejudicial and can hamper the progress of the healing process [4]. Our findings showed that antibiotic use promotes a reduction in the number of inflammatory cells and, therefore, probably favors the transition from the inflammatory to the proliferative phase. In addition, we also observed greater tissue re-epithelialization following the use of antibiotics, which favors cellular proliferation, mainly of fibroblasts and consequently the synthesis of extracellular matrix components. Another important finding was the increase in tissue force following the administration of antibiotics, possibly due to the greater percentage and organization of collagen synthesized by the fibroblasts when compared to the control groups. Therefore, in general, antibiotics are an interesting alternative for wound treatment, given that they favor the transition from the inflammatory to the proliferative phase, the closure of the wound surface, the formation of granulation tissue and increased resistance of the scar.

In recent years, advances in understanding through research related to wound healing has led to the development of different therapies, in search of lower costs together with patient wellbeing [58,59]. Traditional treatments, such as bandages, cotton wool and gauzes only provide initial mechanical protection, but certain characteristics restrict their use, such as their absorbent properties and permeability. This means the wound becomes an environment favorable to the growth of pathogenic microorganisms, in addition to adhering to the surface of the wound, which can induce epithelial trauma during removal [58]. In this context, the use of antibiotic therapies is common and necessary to slow the proliferation of pathogenic microorganisms that can invade the tissue and cause infections, leading to an excessive inflammatory response that could cause chronification of the healing process [59]. Our results showed that antibiotic use, mainly in a curative application based on hydrogel formulations, are important to speed up cutaneous healing. This type of therapy is growing, probably seeking to overcome problems related to traditional treatments, in addition to giving the wound a moist and hydrated surface, ideal for accelerating the process of cutaneous repair.

The findings of our study show that the materials used for the development of new cures for wounds were based on synthetic polymers, cellulose, and gelatin, amongst others. In the majority of the studies where a cure was developed in association with antibiotics, improvements in the tissue repair process were observed. However, to determine if the antibiotic contributed to the positive outcomes of the cure, an adequate control group should have been implemented. The control group needed to present the same elements as the experimental group, except for the evaluated compound [60], in this case, antibiotics. It is interesting to note that few studies reported the presence of a control group with these characteristics, suggesting a need to improve methodological standards in studies investigating the use of antibiotics in healing. The absence of this group is considered a serious methodological failing, since it leads to the obtainment of false-positive results, as well as hampering the determination of the beneficial effects of the substance of interest [61].

Our revision had as its object studies employing animal models, and one of the biggest advantages of the use of these models for wound healing was that they allow for histological monitoring of the wound healing process. Additionally, they allow for the realization of macroscopic, biochemical and biomechanical measurements [62]. In our research, the animal most commonly used in the studies was the rat, possibly due to the low cost, and easy handling and accessibility, allowing researchers to use a relatively large number of animals for their experiments, thereby generating a greater degree of reliability in the results [63]. In addition, the area of dorse is higher when compared to mice. Pig models were the second most used animal, possibly due to similarities between pig and human skin, mainly in terms of general structure, thickness, follicle content, capillaries, pigmentation, collagen content and lipid composition [64]. Some studies used larger animals, however, these studies used a smaller number of animals per group, possibly as a result of the cost, due to its not being practical for the majority of research installations, as well as presenting difficulties with handling during the realization of procedures [65]. In this context, the reduction in the number of experimental animals may be a complicating factor in research due to increased risk of obtaining inconclusive results [66].

Considering wound models, we observed that despite studies that evaluated wounds by incision being included, the greater majority used the excision model when investigating the tissue repair process. The excisional wounds are valid and reproducible models, being very useful in the analysis of healing [67]. In addition to the wound model, the number of wounds per animal is also an important parameter when dealing with studies of healing. In our revision, we found that a considerable number of wounds were made on the same animal, and consequently, these wounds were not located on the same bodily region. The same location for biopsy should be maintained between the groups due to wound contraction, re-epithelialization rates, and total tissue repair varying depending on location [68]. Additionally, the realization of many wounds can increase stress on the animal, and independent of the duration of the stress factor, lead to alterations in local chemical mediators and of the cells involved in the initial stages of wound healing, thereby compromising the results [68].

The class of antibiotics most commonly used in the studies in this revision was sulfonamide, represented by silver sulfadiazine. The prevalence of this antibiotic amongst these studies can be explained by its extensive medical use, being the most widely used topical antimicrobial for burns in recent decades [69]. Topical antibacterial agents, specifically silver sulfadiazine cream, are used to reduce bacterial count. To the contrary of other means of application, the topical route allows the antibiotic to penetrate adequately into the open and granulous wound, being able to exercise a direct bacteriostatic or bactericidal effect on an ample spectrum of gram-positive and gram-negative organisms [70]. Therefore, topical use allows the antibiotic to reach high local concentrations of the drug with minimal systemic absorption, thereby minimizing the risk of adverse systemic effects. Oral administration was performed in two studies by dissolving the pharmacological agent in the animal’s drinking water. This form of administration limits the analysis since it does not precisely determine the quantity of antibiotic ingested by the animal. Additionally, given that no calculation of water consumption was carried out, these studies cannot be reproduced, due to the average consumption of the medication being unknown. Furthermore, this lack of control represents a methodological failing, since it hampers the attribution of the results derived directly from antibiotic use. Administration via the intravenous route, on the other hand, was reported for only one study. This route allows the antibiotic to be distributed throughout the organism, meaning that all wounds on the animal receive the medication, including the control wound when made on the same animal [53].

In the last years, advances in understanding regarding wound healing has led to the development of innumerable therapies, but the enormous costs associated with this disease and the seeks to promote patient well-being have driven new research efforts to find the ideal treatment. As a consequence, a variety of new preparations, curative materials and advanced methods of debridement have been presented in scientific articles through experiments involving animal models. Therefore, this revision brought together published studies that used animal models to investigate antibiotic use in the repair of incisional and excisional wound models. In terms of the methodological analyses performed for these studies, generally the time for wound closure, tissue resistance and histomorphology were evaluated (quantification of matrix components, neovascularization, cells from the lesion area). The results showed that antibiotics led to a reduction in wound area, and a consequent increase in proliferation of fibroblasts and of extracellular matrix components, and an increase in collagen organization, giving the wound greater resistance. Neovascularization was found to be variable between the studies, with molecular analysis being necessary to understand the action of these antibiotics on the regulation of angiogenesis. The methodology used to analyze tissue repair in the majority of the studies was considered poor due to not investigating the molecular or biochemical effects on healing, for example, the action of cytokines and oxidative stress, in addition to poorly explaining the mechanisms involved in wound healing.

The results found in this review showed that antibiotic therapy favors cutaneous healing processes by reducing inflammation and increasing cell and vessel proliferation. However, it is important to highlight that the preventive use of antibiotics should be considered with caution. In the age of multiple drug resistance for antibiotics, the preventive use of antibiotics for treatment of cutaneous lesions is not advisable. Nonetheless, we know that antibiotic therapy is indicated as an adjunct to wound care as it promotes infection control in open wounds, burns and trauma. In this context, antibiotic use has a beneficial effect on the wound healing process, but with the aim of assisting other drugs that are elective to accelerate closure and recover matrix components. Therefore, we believe that antibiotic therapy should be used to treat wounds when there is a risk of infection and not as an elective therapy for treatment of skin lesions. This is important to avoid the natural selection and multiplication of resistant bacteria [71]. Bacterial resistance increases treatment costs, prolongs hospital stays and may consequently increase mortality rates [72]. Therefore, we believe that the rational use of antibiotics and the continued development of alternative treatments are necessary to decrease antibiotic resistance. Among the alternative therapies available today to maximize the effect of antibiotics, or even decrease their use, are immunity modulating agents, bacteriophages and their lysines, antimicrobial peptides, pro and pre-symbiotics, plant extracts, pathogenicity inhibitors (quorum detection bacterial, biofilm and virulence), food enzymes [73], phytochemicals and metals [74].

### Guide to reporting relevant information

Based on the findings of this review, we have created a guide that includes a list of elements that should be reported, quantitatively, in order to make it easier to compare results between articles that test the effects of antibiotics on cutaneous wound healing (S5 Table).

### Limitations

Systematic revisions are considered high level studies that allow for the individual evaluation of studies in a blind manner using specific tools [75]. Such characteristics lead to a more inclusive, non-tendentious approach, to provide the reader with a broad understanding of the studies included in systematic revisions. One of the limitations was that this systematic review has not been registered online, once the register it is an important initiative to promote the quality of scientific publication, promote transparency, reduce duplication, and minimize bias in reviews. The results presented in this study are important and valuable to manage antibiotic use in cutaneous wound treatment. However, they should also be treated with caution, since the majority of the studies presented significant methodological variability, mainly in terms of the control and association or not of other compounds, hampering comparison and categorical conclusions regarding the benefits of antibiotic interventions.

The differences between the days for the realization of the biopsy, the use of different animals and the highly variable wound sizes between studies, meant that the days for wound closure were not comparable between the studies included in this revision. An important observation was that a large number of the studies did not demonstrate the realization of a statistical analysis of their data, reducing the reliability of the results they presented. Information regarding the concentration of the antibiotic administered was also neglected in the studies, with this data being of extreme importance, for example helping to explain why studies that used the same antibiotic presented differing results.

The discrepancies between the studies become clear when we take into consideration simple information such as age, weight, total number of animals and number of wounds per animal. Additionally, the absence of information regarding potential factors of confusion (for example, age, sex, concentration of medication) can lead to erroneous results. Therefore, our evaluation of the risk of bias and methodological quality show that many studies inadequately present their methodology, resulting in high risk of bias. Surprisingly, we did not find a direct relationship between the high methodological bias and the year of publication of the studies, that is, there has possibly been a systematic reproduction of methodological errors over the years, since the quality of the reports has not improved. These findings show the urgent need to develop controlled and randomized studies that aim to reduce biases in the selection, preparation and writing of scientific reports. Therefore, we expect that this revision will be used as a guide to improve reporting for future research into wound treatment with antibiotics.

## Conclusion

The healing of cutaneous wounds has been widely investigated by researchers, being a fundamental area of research due to the considerable functional and aesthetic role of this tissue. When skin is injured, bacteria can infiltrate and colonize the surrounding tissue, which can lead to potentially fatal infections. Therefore, efficient treatment is necessary to control such pathological conditions.

An awareness of the effects of antibiotic treatments in healing of not intentionally infected wounds allows us to understand their influence on the flora and innate immunity of the skin, thereby, giving us a better understanding of the results expected from their use. In our revision, a large percentage of the treatments with antibiotics showed that they were effective in speeding up the healing process for wounds, given that a reduction in the infiltration of inflammatory cells, as well as an increase in the number of fibroblasts and extracellular matrix components and consequently a more rapid and effective closure of cutaneous wounds, was observed. However, the fragility of the current studies was evident, given that the majority presented high risk of bias, which impeded the reproducibility of most of the studies considered in this review.

## Supporting information

S1 Table PRISMA 2009- Checklist of items to include when reporting a systematic review. (DOC)

S2 Table Complete search strategy with search filters and number of studies recovered in databases PubMed-Medline and Scopus. (DOCX)

S3 Table General characteristics of the experimental models used in all studies included in this systematic review. (DOCX)

S4 Table General characteristics of the wounds treated with antibiotics used in all studies included in this systematic review. (DOCX)

S5 Table Guide for relevant information in studies with antibiotic therapy and wound healing. (DOCX)
