# Supplementary material for: Does antibiotic use accelerate or retard cutaneous repair? A systematic review in animal models
Source: PLoS One. 2019 Oct 10;14(10):e0223511. doi: 10.1371/journal.pone.0223511 (PMC6786583; doi:10.1371/journal.pone.0223511)
Supplement: S2 Table — (DOCX) [file pone.0223511.s002.docx]

**Supporting information**

**S2 Table.** Complete search strategy with search filters and number of studies recovered in databases PubMed-Medline and Scopus.

| ***PubMed-MEDLINE - Search Filters*** | ***Number of records*** |
| --- | --- |
| **#1 Antibiotic**  ("anti-bacterial agents"[MeSH Terms] OR “anti-bacterial agents”[TIAB] OR “antibiotic”[TIAB] OR “Anti Bacterial Agents”[TIAB] OR “Antibacterial Agents”[TIAB] OR “antibacterial”[TIAB] OR “Anti-Bacterial Compounds”[TIAB] OR “Anti Bacterial Compounds”[TIAB] OR “Bacteriocidal Agents”[TIAB] OR “Bacteriocides”[TIAB] OR “Anti-Mycobacterial Agents”[TIAB] OR “Anti Mycobacterial Agents”[TIAB] OR “Antimycobacterial Agents”[TIAB] OR “Antibiotics”[TIAB] OR “anti-biotic”[TIAB] OR "anti-infective agents"[MeSH Terms] OR “anti-infective agents”[TIAB] OR “Anti Infective Agents”[TIAB] OR “Antiinfective Agents”[TIAB] OR “antiinfective”[TIAB] OR “Microbicides”[TIAB] OR “Antimicrobial Agents”[TIAB] OR “Anti-Microbial Agents”[TIAB] OR “Anti Microbial Agents”[TIAB] OR “anti-microbial”[TIAB] OR “anti-infective”[TIAB] OR “antibiotic therapy”[TIAB]) | **826371** |
| **#2 Wound Healing**  (“Wound Healing”[MeSH terms] OR “Regeneration”[MeSH terms] OR “Wound Healing”[TIAB] OR “Regeneration”[TIAB]) | **297911** |
| **#3 Skin**  (“Skin”[MeSH terms] OR “Dermis”[MeSH terms] OR “Granulation Tissue”[MeSH terms] OR “Epidermis”[MeSH terms] OR “Keratinocytes”[MeSH terms] OR “Integumentary System”[MeSH terms] OR “Dermatology”[MeSH terms] OR “Dermoscopy”[MeSH terms] OR “Wounds and Injuries”[MeSH terms] OR “Fibrosis”[MeSH terms] OR “Skin injuries”[TIAB] OR “Skin fibrosis”[TIAB] OR “Skin scars”[TIAB] OR “Skin cicatriz”[TIAB] OR “Cicatrix”[MeSH terms] OR “cutaneous”[TIAB) | **1262536** |
| **#4 First animal filter***  ("animal experimentation"[MeSH Terms] OR "models, animal"[MeSH Terms] OR "invertebrates"[MeSH Terms] OR "Animals"[Mesh:noexp] OR "animal population groups"[MeSH Terms] OR "chordata"[MeSH Terms:noexp] OR "chordata, nonvertebrate"[MeSH Terms] OR "vertebrates"[MeSH Terms:noexp] OR "amphibians"[MeSH Terms] OR "birds"[MeSH Terms] OR "fishes"[MeSH Terms] OR "reptiles"[MeSH Terms] OR "mammals"[MeSH Terms:noexp] OR "primates"[MeSH Terms:noexp] OR "artiodactyla"[MeSH Terms] OR "carnivora"[MeSH Terms] OR "cetacea"[MeSH Terms] OR "chiroptera"[MeSH Terms] OR "elephants"[MeSH Terms] OR "hyraxes"[MeSH Terms] OR "insectivora"[MeSH Terms] OR "lagomorpha"[MeSH Terms] OR "marsupialia"[MeSH Terms] OR "monotremata"[MeSH Terms] OR "perissodactyla"[MeSH Terms] OR "rodentia"[MeSH Terms] OR "scandentia"[MeSH Terms] OR "sirenia"[MeSH Terms] OR "xenarthra"[MeSH Terms] OR "haplorhini"[MeSH Terms:noexp] OR "strepsirhini"[MeSH Terms] OR "platyrrhini"[MeSH Terms] OR "tarsii"[MeSH Terms] OR "catarrhini"[MeSH Terms:noexp] OR "cercopithecidae"[MeSH Terms] OR "hylobatidae"[MeSH Terms] OR "hominidae"[MeSH Terms:noexp] OR "gorilla gorilla"[MeSH Terms] OR "pan paniscus"[MeSH Terms] OR "pan troglodytes"[MeSH Terms] OR "pongo pygmaeus"[MeSH Terms]) | **6308321** |
| **#5 Second animal filter***  ("animals"[TIAB] OR "animal"[TIAB] OR "mice"[TIAB] OR "mus"[TIAB] OR "mouse"[TIAB] OR "murine"[TIAB] OR "woodmouse"[TIAB] OR "rats"[TIAB] OR "rat"[TIAB] OR "murinae"[TIAB] OR "muridae"[TIAB] OR "cottonrat"[TIAB] OR "cottonrats"[TIAB] OR "hamster"[TIAB] OR "hamsters"[TIAB] OR "cricetinae"[TIAB] OR "rodentia"[TIAB] OR "rodent"[TIAB] OR "rodents"[TIAB] OR "pigs"[TIAB] OR "pig"[TIAB] OR "swine"[TIAB] OR "swines"[TIAB] OR "piglets"[TIAB] OR "piglet"[TIAB] OR "boar"[TIAB] OR "boars"[TIAB] OR "sus scrofa"[TIAB] OR "ferrets"[TIAB] OR "ferret"[TIAB] OR "polecat"[TIAB] OR "polecats"[TIAB] OR "mustela putorius"[TIAB] OR "guinea pigs"[TIAB] OR "guinea pig"[TIAB] OR "cavia"[TIAB] OR "callithrix"[TIAB] OR "marmoset"[TIAB] OR "marmosets"[TIAB] OR "cebuella"[TIAB] OR "hapale"[TIAB] OR "octodon"[TIAB] OR "chinchilla"[TIAB] OR "chinchillas"[TIAB] OR "gerbillinae"[TIAB] OR "gerbil"[TIAB] OR "gerbils"[TIAB] OR "jird"[TIAB] OR "jirds"[TIAB] OR "merione"[TIAB] OR "meriones"[TIAB] OR "rabbits"[TIAB] OR "rabbit"[TIAB] OR "hares"[TIAB] OR "hare"[TIAB] OR "diptera"[TIAB] OR "flies"[TIAB] OR "fly"[TIAB] OR "dipteral"[TIAB] OR "drosphila"[TIAB] OR "drosophilidae"[TIAB] OR "cats"[TIAB] OR "cat"[TIAB] OR "carus"[TIAB] OR "felis"[TIAB] OR "nematoda"[TIAB] OR "nematode"[TIAB] OR "nematoda"[TIAB] OR "nematode"[TIAB] OR "nematodes"[TIAB] OR "sipunculida"[TIAB] OR "dogs"[TIAB] OR "dog"[TIAB] OR "canine"[TIAB] OR "canines"[TIAB] OR "canis"[TIAB] OR "sheep"[TIAB] OR "sheeps"[TIAB] OR "mouflon"[TIAB] OR "mouflons"[TIAB] OR "ovis"[TIAB] OR "goats"[TIAB] OR "goat"[TIAB] OR "capra"[TIAB] OR "capras"[TIAB] OR "rupicapra"[TIAB] OR "chamois"[TIAB] OR "haplorhini"[TIAB] OR "monkey"[TIAB] OR "monkeys"[TIAB] OR "anthropoidea"[TIAB] OR "anthropoids"[TIAB] OR "saguinus"[TIAB] OR "tamarin"[TIAB] OR "tamarins"[TIAB] OR "leontopithecus"[TIAB] OR "hominidae"[TIAB] OR "ape"[TIAB] OR "apes"[TIAB] OR "pan"[TIAB] OR "paniscus"[TIAB] OR "pan paniscus"[TIAB] OR "bonobo"[TIAB] OR "bonobos"[TIAB] OR "troglodytes"[TIAB] OR "pan troglodytes"[TIAB] OR "gibbon"[TIAB] OR "gibbons"[TIAB] OR "siamang"[TIAB] OR "siamangs"[TIAB] OR "nomascus"[TIAB] OR "symphalangus"[TIAB] OR "chimpanzee"[TIAB] OR "chimpanzees"[TIAB] OR "prosimians"[TIAB] OR "bush baby"[TIAB] OR "prosimian"[TIAB] OR "bush babies"[TIAB] OR "galagos"[TIAB] OR "galago"[TIAB] OR "pongidae"[TIAB] OR "gorilla"[TIAB] OR "gorillas"[TIAB] OR "pongo"[TIAB] OR "pygmaeus"[TIAB] OR "pongo pygmaeus"[TIAB] OR "orangutans"[TIAB] OR "pygmaeus"[TIAB] OR "lemur"[TIAB] OR "lemurs"[TIAB] OR "lemuridae"[TIAB] OR "horse"[TIAB] OR "horses"[TIAB] OR "pongo"[TIAB] OR "equus"[TIAB] OR "cow"[TIAB] OR "calf"[TIAB] OR "bull"[TIAB] OR "chicken"[TIAB] OR "chickens"[TIAB] OR "gallus"[TIAB] OR "quail"[TIAB] OR "bird"[TIAB] OR "birds"[TIAB] OR "quails"[TIAB] OR "poultry"[TIAB] OR "poultries"[TIAB] OR "fowl"[TIAB] OR "fowls"[TIAB] OR "reptile"[TIAB] OR "reptilia"[TIAB] OR "reptiles"[TIAB] OR "snakes"[TIAB] OR "snake"[TIAB] OR "lizard"[TIAB] OR "lizards"[TIAB] OR "alligator"[TIAB] OR "alligators"[TIAB] OR crocodile[TIAB] OR "crocodiles"[TIAB] OR "turtle"[TIAB] OR "turtles"[TIAB] OR "amphibian"[TIAB] OR "amphibians"[TIAB] OR "amphibia"[TIAB] OR "frog"[TIAB] OR "frogs"[TIAB] OR "bombina"[TIAB] OR "salientia"[TIAB] OR "toad"[TIAB] OR "toads"[TIAB] OR "epidalea calamita"[TIAB] OR "salamander"[TIAB] OR "salamanders"[TIAB] OR "eel"[TIAB] OR "eels"[TIAB] OR "fish"[TIAB] OR "fishes"[TIAB] OR "pisces"[TIAB] OR "catfish"[TIAB] OR "catfishes"[TIAB] OR "siluriformes"[TIAB] OR "arius"[TIAB] OR "heteropneustes"[TIAB] OR "sheatfish"[TIAB] OR "perch"[TIAB] OR "perches"[TIAB] OR "percidae"[TIAB] OR "perca"[TIAB] OR "trout"[TIAB] OR "trouts"[TIAB] OR "char"[TIAB] OR "chars"[TIAB] OR "salvelinus"[TIAB] OR "fathead minnow"[TIAB] OR "minnow"[TIAB] OR "cyprinidae"[TIAB] OR "carps"[TIAB] OR "carp"[TIAB] OR "zebrafish"[TIAB] OR "zebrafishes"[TIAB] OR "goldfish"[TIAB] OR "goldfishes"[TIAB] OR "guppy"[TIAB] OR "guppies"[TIAB] OR "chub"[TIAB] OR "chubs"[TIAB] OR "tinca"[TIAB] OR "barbels"[TIAB] OR "barbus"[TIAB] OR "pimephales"[TIAB] OR "promelas"[TIAB] OR "poecilia reticulata"[TIAB] OR "mullet"[TIAB] OR "mullets"[TIAB] OR "seahorse"[TIAB] OR "seahorses"[TIAB] OR "mugil curema"[TIAB] OR "atlantic cod"[TIAB] OR "shark"[TIAB] OR "sharks"[TIAB] OR "catshark"[TIAB] OR "anguilla"[TIAB] OR "salmonid"[TIAB] OR "salmonids"[TIAB] OR "whitefish"[TIAB] OR "whitefishes"[TIAB] OR "salmon"[TIAB] OR "salmons"[TIAB] OR "sole"[TIAB] OR "solea"[TIAB] OR "sea lamprey"[TIAB] OR "lamprey"[TIAB] OR "lampreys"[TIAB] OR "pumpkinseed"[TIAB] OR "sunfish"[TIAB] OR "sunfishes"[TIAB] OR "tilapia"[TIAB] OR "tilapias"[TIAB] OR "turbot"[TIAB] OR "turbots"[TIAB] OR "flatfish"[TIAB] OR "flatfishes"[TIAB] OR "sciuridae"[TIAB] OR "squirrel"[TIAB] OR "squirrels"[TIAB] OR "chipmunk"[TIAB] OR "chipmunks"[TIAB] OR "suslik"[TIAB] OR "susliks"[TIAB] OR "vole"[TIAB] OR "voles"[TIAB] OR "lemming"[TIAB] OR "lemmings"[TIAB] OR "muskrat"[TIAB] OR "muskrats"[TIAB] OR "lemmus"[TIAB] OR "otter"[TIAB] OR "otters"[TIAB] OR "marten"[TIAB] OR "martens"[TIAB] OR "martes"[TIAB] OR "weasel"[TIAB] OR "badger"[TIAB] OR "badgers"[TIAB] OR "ermine"[TIAB] OR "mink"[TIAB] OR "minks"[TIAB] OR "sable"[TIAB] OR "sables"[TIAB] OR "gulo"[TIAB] OR "gulos"[TIAB] OR "wolverine"[TIAB] OR "wolverines"[TIAB] OR "minks"[TIAB] OR "mustela"[TIAB] OR "llama"[TIAB] OR "llamas"[TIAB] OR "alpaca"[TIAB] OR "alpacas"[TIAB] OR "camelid"[TIAB] OR "camelids"[TIAB] OR "guanaco"[TIAB] OR "guanacos"[TIAB] OR "chiroptera"[TIAB] OR "chiropteras"[TIAB] OR "bat"[TIAB] OR "bats"[TIAB] OR "fox"[TIAB] OR "foxes"[TIAB] OR "iguana"[TIAB] OR "iguanas"[TIAB] OR "xenopus laevis"[TIAB] OR "parakeet"[TIAB] OR "parakeets"[TIAB] OR "parrot"[TIAB] OR "parrots"[TIAB] OR "donkey"[TIAB] OR "donkeys"[TIAB] OR "mule"[TIAB] OR "mules"[TIAB] OR "zebra"[TIAB] OR "zebras"[TIAB] OR "shrew"[TIAB] OR "shrews"[TIAB] OR "bison"[TIAB] OR "bisons"[TIAB] OR "buffalo"[TIAB] OR "buffaloes"[TIAB] OR "deer"[TIAB] OR "deers"[TIAB] OR "bear"[TIAB] OR "bears"[TIAB] OR "panda"[TIAB] OR "pandas"[TIAB] OR "wild hog"[TIAB] OR "wild boar"[TIAB] OR "fitchew"[TIAB] OR "fitch"[TIAB] OR "beaver"[TIAB] OR "beavers"[TIAB] OR "jerboa"[TIAB] OR "jerboas"[TIAB] OR "capybara"[TIAB] OR "capybaras"[TIAB]] NOT "medline"[SUBSET]) | **322580** |
| **Combined search:** ((((#5) OR #4) AND #3) AND #2) AND #1 | **934** |
|  |  |
| ***SCOPUS - Search Filters*** | ***Retrieved records*** |
| **#1 Antibiotic**  (TITLE-ABS-KEY ("anti-bacterial agents") OR TITLE-ABS-KEY (“anti-bacterial agents”) OR TITLE-ABS-KEY (“antibiotic”) OR TITLE-ABS-KEY (“Anti Bacterial Agents”) OR TITLE-ABS-KEY (“Antibacterial Agents”) OR TITLE-ABS-KEY (“antibacterial”) OR TITLE-ABS-KEY (“Anti-Bacterial Compounds”) OR TITLE-ABS-KEY (“Anti Bacterial Compounds”) OR TITLE-ABS-KEY (“Bacteriocidal Agents”) OR TITLE-ABS-KEY (“Bacteriocides”) OR TITLE-ABS-KEY (“Anti-Mycobacterial Agents”) OR TITLE-ABS-KEY (“Anti Mycobacterial Agents”) OR TITLE-ABS-KEY (“Antimycobacterial Agents”) OR TITLE-ABS-KEY (“Antibiotics”) OR TITLE-ABS-KEY (“anti-biotic”) OR TITLE-ABS-KEY (“anti-infective agents”) OR TITLE-ABS-KEY (“anti-infective agents”) OR TITLE-ABS-KEY (“Anti Infective Agents”) OR TITLE-ABS-KEY (“Antiinfective Agents”) OR TITLE-ABS-KEY (“antiinfective”) OR TITLE-ABS-KEY (“Microbicides”) OR TITLE-ABS-KEY (“Antimicrobial Agents”) OR TITLE-ABS-KEY (“Anti-Microbial Agents”) OR TITLE-ABS-KEY (“Anti Microbial”) Agents OR TITLE-ABS-KEY (“anti-microbial”) OR TITLE-ABS-KEY (“anti-infective”) OR TITLE-ABS-KEY (“antibiotic therapy”)) | **777074** |
| **#2 Wound Healing**  (TITLE-ABS-KEY (“wound healing”) OR TITLE-ABS-KEY (“Regeneration”)) | **424226** |
| **#3 Skin**  TITLE-ABS-KEY(Skin) OR TITLE-ABS-KEY(Dermis) OR TITLE-ABS-KEY(“Granulation Tissue”) OR TITLE-ABS-KEY(Epidermis) OR TITLE-ABS-KEY(Keratinocyte*) OR TITLE-ABS-KEY(Integumentary System) OR TITLE-ABS-KEY(Dermatology) OR TITLE-ABS-KEY(Dermoscopy) OR TITLE-ABS-KEY(Skin wounds) OR TITLE-ABS-KEY(Skin injuries) OR TITLE-ABS-KEY(Skin fibrosis) OR TITLE-ABS-KEY(Skin scar*) OR (Skin cicatrix) OR (cutaneous))injuries”) OR TITLE-ABS-KEY(“skin fibrosis”) OR TITLE-ABS-KEY(“skin scars”)) | **1590396** |
| **Combined search:** #1 AND #2 AND #3  (Limited to experimental animals and English, Portuguese and Spanish language) | **602** |

* In PubMed-Medline database, standardized animal filters were obtained in Hooijmans, et al. [25].
