# Supplementary material for: Does antibiotic use accelerate or retard cutaneous repair? A systematic review in animal models
Source: PLoS One. 2019 Oct 10;14(10):e0223511. doi: 10.1371/journal.pone.0223511 (PMC6786583; doi:10.1371/journal.pone.0223511)
Supplement: S3 Table — (DOCX) [file pone.0223511.s003.docx]

**Supporting Information**

**S3 Table**. General characteristics of the experimental models used in all studies included in this systematic review

| **Animal model: Rat** | | | | | | | | |  |
| --- | --- | --- | --- | --- | --- | --- | --- | --- | --- |
| **Reference** | **Country** | **Strain** | **Sex** | **Age** | **Weight** | **Total number** | **Ethics committee** | **Statistic test** | |
| Leitch, et al. 1993 [27] | AUS | Sprague-Dawley | M | ? | 250–300 g | 40 | Yes | Yes | |
| Heggers et al. 1995 [28] | USA | SpragueDawley | ? | ? | ? | 60 | ? | Yes | |
| Choi et al. 1999 [29] | KOR | Wistar | ? | ? | 180—200 g | ? | ? | No | |
| Muller et al. 2003 [30] | USA | SpragueDawley | M | ? | 250–300 g | 70 | Yes | Yes | |
| Kim, et al. 2008 [31] | KOR | Sprague‐Dawley | M | ? | 250—300 g | ? | ? | No | |
| Kim, et al. 2008 [32] | KOR | SpragueDawley | M | ? | 250–300 g | ? | ? | No | |
| Simpson, et al. 2008 [33] | USA | Sprague‐Dawley | F | Adult (± 8 wk) | 250–300 g | 32 | Yes | Yes | |
| Hwang et al. 2010 [34] | KOR | SpragueDawley | ? | ? | 250–280 g | 10 | Yes | Yes | |
| Lin et al. 2010 [35] | TWN | SpragueDawley | M | 8 wk | 250–300 g | 10 | Yes | Yes | |
| Huang et al. 2012 [36] | TUR | SpragueDawley | F | 4 mo | 200–250 g | 10 | Yes | Yes | |
| Gurel et al. 2013 [37] | IND | Sprague‐Dawley | F | ? | 220–250 g | ? | Yes | Yes | |
| Mittal and Kumar 2014 [38] | IND | Wistar | M | ? | ? | ? | ? | No | |
| Princely et al. 2015 [39] | CHN | SpragueDawley | ? | ? | 200–250 g | 12 | Yes | Yes | |
| Fu et al. 2016 [40] | CHN | Sprague‐Dawley | M | ? | 220–250 g | 35 | Yes | No | |
| **Animal model: Pig** | | | | | | | | |  |
| **Reference** | **Country** | **Strain** | **Sex** | **Age** | **Weight** | **Total number** | **Ethics committee** | **Statistic test** | |
| Geronemus, et al. 1979 [42] | USA | ? | ? | Young (± 3 yr) | 5.5–9.1 kg | 17 | ? | Yes | |
| Watcher and Wheeland 1989 [43] | USA | Minipig | ? | Young (± 3 yr) | 10.5/ 13.0 kg | 2 | ? | Yes | |
| Singer, et al. 1999 [44] | USA | Yorkshire | F | ? | 20–30 kg | 3 | Yes | Yes | |
| Faucher, et al. 2010 [45] | USA | Yucatan | ? | ? | 18kg | 8 | Yes | Yes | |
| Theunissen et al. 2016 [46] | ZAF | ? | F | ? | 5–20 kg | 16 | Yes | Yes | |
| **Animal model: Mice** |  |  |  |  |  |  |  |  | |
| **Reference** | **Country** | **Strain/ Animal** | **Sex** | **Age** | **Weight** | **Total number** | **Ethics committee** | **Statistic test** | |
| Hebda et al. 2003 [47] | USA | FVB | ? | ? | ? | 16/24 | Yes | Yes | |
| Zhang et al. 2015 [48] | TWN | C57BL/6J | M | 8–10 wk | ? | 8 | Yes | Yes | |
| Tummalapalli et al. 2016 [49] | IND | C57BL/6J | M | 10 wk | 20–25 g | 25 | Yes | Yes | |
| Kataria et al. 2014 [50] | IND | ? | M | ? | 1.5–3 kg | 12 | Yes | No | |
| Qian, et al. 2017 [51] | USA | New Zealand | F | Adult (± 6 mo) | 3–5 kg | 24 | Yes | Yes | |
| Berry and Sullins 2003 [52] | USA | Thoroughbreds, Warmblood and Quarter Horse | M | 5–11 yr | 527–645 kg | 6 | Yes | Yes | |
| Edwards-Milewski et al. 2016 [53] | USA | Quarter Horses and Thoroughbreds | M/F | 6–23 yr | 402–581 kg | 7 | Yes | Yes | |

AUS = Australia, CHN = China, d = day, F = female, IND = India, KOR = South Korea, M = male, mo = month, TUR = Turkey, TWN = Taiwan, USA = United States of America, wk = week, yr = year, ZAF = South Africa.
