# Supplementary material for: Does antibiotic use accelerate or retard cutaneous repair? A systematic review in animal models
Source: PLoS One. 2019 Oct 10;14(10):e0223511. doi: 10.1371/journal.pone.0223511 (PMC6786583; doi:10.1371/journal.pone.0223511)
Supplement: S4 Table — (DOCX) [file pone.0223511.s004.docx]

**Supporting Information**

**Table S4**. General characteristics of the wounds treated with antibiotics used in all studies included in this systematic review

| **Wound** | | | | | | |
| --- | --- | --- | --- | --- | --- | --- |
| **Animal model: Rat** | | | | | | |
| **Reference** | **Asepxis** | **Biopsia day** | **Realization** | **Size** | **Number for animal** | **Anesthesia (drug and dose)** |
| Leitch, et al. 1993 [27] | ? | ? | ? | 2.25 cm^2^ | 4 | Iso (?) |
| Heggers et al. 1995 [28] | ? | ? | ? | 1.5 cm^2^ | 4 | ? |
| Choi et al. 1999 [29] | PI | 5/12 | ? | 1 cm (Diam) | ? | Thiopental (?) |
| Muller et al. 2003 [30] | ? | NA | ? | 2.25 cm^2^ | 4 | Pentobarbital (?) |
| Kim, et al. 2008 [31] | EtOH 70% | 5/15 | ? | 2.25 cm^2^ | 2 | Tilet + Zolaz (?)/ Xyl (?) |
| Kim, et al. 2008 [32] | EtOH 70% | 5/ 10/ 15 | ? | 2.25 cm^2^ | 2 | Tilet (125,0 mg) + Zolaz (125,0 mg) / Xyl (20 mg/mL) |
| Simpson, et al. 2008 [33] | CHX 4%/ sal | 4/ 8/ 14 | ? | 8 mm (Diam) | 2 | Iso (1.25–2%) |
| Hwang et al. 2010 [34] | EtOH 70% | 15 | ? | 2.25 cm^2^ | 2 | Tilet (125,0 mg) + Zolaz (125,0 mg) |
| Lin et al. 2010 [35] | EtOH 70% | NA | ? | 1 cm^2^ | 2 | Ket (90 mg/kg)/ Xyl (10 mg/kg) |
| Huang et al. 2012 [36] | EtOH 70% | 2/ 4/ 8/ 12 | ? | 1 cm^2^ | ? | Ket (90 mg/kg) + Xyl (10 mg/kg) |
| Gurel et al. 2013 [37] | Sal | 4/ 7/ 14/ 21 | Punch biopsy/ scalpel | 5 mm | 12 | Ket (70 mg/kg) + Cpz (3 mg/kg) |
| Mittal and Kumar 2014 [38] | EtOH 70% | 3/ 7/ 14/ 28/ 45 | ? | 2 cm (Diam) | ? | Ket (80 mg/kg) +Xyl (5 mg/kg) |
| Princely et al. 2015 [39] | ? | NA | Scissor | 2 cm, 2 mm deep | 1 | Ket (50 mg/kg) |
| Fu et al. 2016 [40] | EtOH 75% | 8/ 14 | Scissor | 10 mm (Diam) | 4 | Chloral hydrate (480 mg/kg) |
| Li et al. 2017 [41] | ? | 21 | ? | 9 cm^2^ | 1 | Pentobarbitone (50 mg/kg) |
| **Animal model: Pig** | | | | | | |
| **Reference** | **Asepxis** | **Biopsia day** | **Realization** | **Size** | **Number for animal** | **Anesthesia (drug and dose)** |
| Geronemus, et al. 1979 [42] | ? | 2–7 | Dermatome | 0.7 cm^2^, 0.3 mm deep | 150 | Pentobarbital (15 mg/kg) |
| Watcher and Wheeland 1989 [43] | PI | NA | Scalpel | 4 cm^2^ | 20 | Ket (10 mg/kg) + Halo (?) |
| Singer, et al. 1999 [44] | PI/ sal | 7/ 14/ 30 | Punch biopsy | 4 mm | 36/45 | Tilet + Zolaz (5 mg/kg) / Iso (0.5-2.5%) |
| Faucher, et al. 2010 [45] | PI | 7/ 9/ 14/ 21 | Dermatome | 0.055/ 0.076 cm | 2 | Tilet + Zolaz (4.4 mg/kg) + Xyl (2.2 mg/kg)/ Iso |
| Theunissen et al. 2016 [46] | No | 3/ 7/ 28 | Scalpel | 4 cm^2^ | 16 | Halo (?) |
| **Animal model: Mice** | | | | | | |
| **Reference** | **Asepxis** | **Biopsia day** | **Realization** | **Size** | **Number for animal** | **Anesthesia (drug and dose)** |
| Hebda et al. 2003 [47] | ? | 7/ 14/ 21 | Dermatome/ scissor | 0.7 cm^2^, 0.2–0.3 mm deep/ 1cm (Diam) | 1 | Halo (?) |
| Zhang et al. 2015 [48] | ? | 5 | Punch biopsy | 8 mm | ? | Pentobarbital (?) |
| Tummalapalli et al. 2016 [49] | ? | 21 | Punch biopsy | 8 mm | 1 | Iso (?) |
| **Animal model: Rabbit** | | | | | | |
| **Reference** | **Asepxis** | **Biopsia day** | **Realization** | **Size** | **Number for animal** | **Anesthesia (drug and dose)** |
| Kataria et al. 2014 [50] | ? | 5/ 15 | Scalpel | 4 cm^2^, 0.5 cm deep | 1 | Ket (75 mg/kg) |
| Qian, et al. 2017 [51] | PI/ EtOH 70% | 7/ 28 | ? | 7mm (Diam) | 6 | Ket (22.5 mg/kg) + Xyl (3.5 mg/kg)/ Lid (1%) + Epi |
| **Animal model: Horse** | | | | | | |
| **Reference** | **Asepxis** | **Biopsia day** | **Realization** | **Size** | **Number for animal** | **Anesthesia (drug and dose)** |
| Berry and Sullins 2003 [52] | CHX | NA | Scalpel | 6.25 cm^2^ | 6 | Ket (2.2 mg/kg)/ Xyl (500 mg) + Ket (2 mg) + guaifenesin (50 g) |
| Edwards-Milewski et al. 2016 [53] | CHX 4% / sal/ EtOH | 2/ 5/ 13 | Scalpel | 6.25 cm^2^ | 4 | Detomidine (0.01 mg/kg) + butorphanol tartrate (0.04 mg/kg)/ mepivacaine (2%) |

CHX = chlorhexidine, Cpz = Chlorpromazine, Diam = diameter, Epi = epinephrine, EtOH = ethanol, Halo = Halothane, Iso = Isoflurane, Ket = Ketamine, Lid = lidocaine, NA = Not applicable, PI= povidone-iodine, Sal = saline solution, Tilet = Tiletamine, Xyl = xylazine, Zolaz = Zolazep.
