# Supplementary material for: Does antibiotic use accelerate or retard cutaneous repair? A systematic review in animal models
Source: PLoS One. 2019 Oct 10;14(10):e0223511. doi: 10.1371/journal.pone.0223511 (PMC6786583; doi:10.1371/journal.pone.0223511)
Supplement: S5 Table — (DOCX) [file pone.0223511.s005.docx]

**Supporting Information**

**Table S5.** Guide for relevant information in studies with antibiotic therapy and wound healing.

| General characteristics of experiments | General characteristics of experimental models | Characteristics of antibiotic treatment | Wound characteristics |
| --- | --- | --- | --- |
| 1. Statistical procedure;    2. Sample size (n) for each analysis;  3. Animal ethics committee approval;  4. Appropriate control group (same formulation given for experimental groups, except antibiotic). | 1. Animal model;  2. Strain;  3. Animals age;  4. Average weight of animals;  5. Animals sex;  6. Animals amount per group. | 1. Drug;  2. Pharmaceutical form;  3. Drug concentration;    4. Administered dose;  5. Route of administration;  6. Administration number per period;  7. Treatment duration. | 1. Material used to shave the animal;  2. Information about skin assepsis before surgical procedure;  3. Anesthetic drug;  4. Object used to make the would;  5. Wound area;  6. Would number per animal;  7. Drug used after surgical procedure;  8. Occlusion or lack of wound occlusion;  Wound area assessment periods;  9. Biopsy days; |
|  |  |  |  |
|  |  |  |  |
|  |  |  |  |
|  |  |  |  |
|  |  |  |  |
|  |  |  |  |
|  |  |  |  |
|  |  |  |  |
|  |  |  |  |

Important points that should be reported in antibiotic therapy studies to improve study quality and reports
